# Dietary intervention, but not losartan, completely reverses non-alcoholic steatohepatitis in obese and insulin resistant mice

**DOI:** 10.1186/s12944-017-0432-7

**Published:** 2017-02-23

**Authors:** Jef Verbeek, Pieter Spincemaille, Ilse Vanhorebeek, Greet Van den Berghe, Ingrid Vander Elst, Petra Windmolders, Jos van Pelt, Schalk van der Merwe, Pierre Bedossa, Frederik Nevens, Bruno Cammue, Karin Thevissen, David Cassiman

**Affiliations:** 1 Department of Hepatology, University Hospitals KU Leuven, Leuven, Belgium; 2 Division of Gastroenterology & Hepatology, Department of Internal Medicine, Maastricht University Medical Center, PO box 5800, 6202 AZ Maastricht, The Netherlands; 3 Department of Laboratory Medicine, University Hospitals KU Leuven, Leuven, Belgium; 4 Clinical Department and Laboratory of Intensive Care Medicine, Division Cellular and Molecular Medicine, KU Leuven, Leuven, Belgium; 5 Department of Pathology, Hopital Beaujon, Clichy, France; 6 Centre of Microbial and Plant Genetics (CMPG), KU Leuven, Leuven, Belgium; 7 Department of Plant Systems Biology, Vlaams Instituut voor Biotechnologie (VIB), Ghent, Belgium; 8 Metabolic Center, University Hospitals KU Leuven, Leuven, Belgium

**Keywords:** NASH, treatment, Diet, Losartan, Angiotensin

## Abstract

**Background:**

Dietary intervention is the cornerstone of non-alcoholic steatohepatitis (NASH) treatment. However, histological evidence of its efficacy is limited and its impact on hepatic pathways involved in NASH is underreported. The efficacy of the angiotensin receptor type 1 blocker losartan is controversial because of varying results in a few animal and human studies. We evaluated the effect of dietary intervention versus losartan on NASH and associated systemic metabolic features in a representative mouse model.

**Methods:**

Male C57BL/6 J mice with high fat-high sucrose diet (HF-HSD) induced NASH, obesity, insulin resistance and hypercholesterolemia were subjected to dietary intervention (switch from HF-HSD to normal chow diet (NCD)) (*n* = 9), continuation HF-HSD together with losartan (30 mg/kg/day) (*n* = 9) or continuation HF-HSD only (*n* = 9) for 8 weeks. 9 mice received NCD during the entire experiment (20 weeks). We assessed the systemic metabolic effects and performed a detailed hepatic histological and molecular profiling. A *P*-value of < 0.05, using the group with continuation of HF-HSD only as control, was considered as statistically significant.

**Results:**

Dietary intervention normalized obesity, insulin resistance, and hypercholesterolemia (for all *P* < 0.001), and remarkably, completely reversed all histological features of pre-existent NASH (for all *P* < 0.001), including fibrosis measured by quantification of collagen proportional area (*P* < 0.01). At the hepatic molecular level, dietary intervention targeted fibrogenesis with a normalization of collagen type I alpha 1, transforming growth factor β1, tissue inhibitor of metalloproteinase 1 mRNA levels (for all *P* < 0.01), lipid metabolism with a normalization of fatty acid translocase/CD36, fatty acid transport protein 5, fatty acid synthase mRNA levels (*P* < 0.05) and markers related to mitochondrial function with a normalization of hepatic ATP content (*P* < 0.05) together with sirtuin1 and uncoupling protein 2 mRNA levels (for both *P* < 0.001). Dietary intervention abolished p62 accumulation (*P* < 0.01), suggesting a restoration of autophagic flux. Losartan did not significantly affect obesity, insulin resistance, hypercholesterolemia or any histological NASH feature.

**Conclusions:**

Dietary intervention, and not losartan, completely restores the metabolic phenotype in a representative mouse model with pre-existent NASH, obesity, insulin resistance and hypercholesterolemia.

## Background

Non-alcoholic fatty liver disease (NAFLD) is the most prevalent liver disease in the Western world, affecting 20–30% of the adult population [1]. Due to its strong pathophysiological and epidemiological association with obesity, insulin resistance, dyslipidemia and hypertension, NAFLD is considered as the hepatic manifestation of the metabolic syndrome [2]. NAFLD is the collective noun for a spectrum of histological abnormalities ranging from isolated steatosis to nonalcoholic steatohepatitis (NASH). The latter is characterized by steatosis, hepatocyte ballooning and inflammation with or without fibrosis [3]. NASH can progress to cirrhosis and related complications, including hepatocellular carcinoma [4]. As a consequence, NASH is the most rapidly rising indication for liver transplantation [5, 6].

This societal and medical relevance of NASH has inspired numerous animal studies and human trials in order to find an effective treatment. Dietary modification and physical exercise remain the cornerstones of treatment, although available evidence that these measures can reverse NASH is rather limited [7–11]. When applied together, they may dose-dependently improve the histological features of NASH in a subset of patients, as shown in two clinical trials [8, 11]. Data on the histological effects of diet only interventions on established NASH are even more scarce [10]. However, knowledge of the therapeutic potential of dietary intervention is important, not in the least for patients who cannot increase their level of physical exercise due to physical limitations or lack of time. Furthermore, the mechanisms behind the possible beneficial effects of dietary intervention remain largely underexplored [12].

In addition, not a single pharmacological agent has been approved for the treatment of NASH so far [7, 9, 13]. Research efforts rather improved the insight in NASH pathophysiology, including the role of the renin-angiotensin system (RAS). In the liver, angiotensin II, the main effector of the RAS, is thought to play a role in the development and progression of NASH, possibly by the generation of oxidative stress and inflammatory cytokines leading to steatosis, inflammation and fibrosis [14]. Therefore, attenuating the effects of angiotensin II by administering losartan, an angiotensin II type 1 receptor blocker, could be beneficial for NASH and other components of the metabolic syndrome [14]. However, in vivo evidence regarding the efficacy of losartan is only based on a few studies in non-physiological animal models and two human pilot trials with each a limited number of patients [14–17]. The lack of efficacy of losartan in other studies, further fueled the controversy [18, 19].

Previously, we developed a NASH mouse model induced by the administration of a ‘Western’ high fat-high sucrose diet (HF-HSD), with close resemblance to the hepatic and systemic metabolic profile of typical NASH patients [20]. In the current study, we assessed the therapeutic efficacy of dietary intervention (based on a switch from HF-HSD to normal chow diet) and losartan in this mouse model with pre-existent NASH, insulin resistance, obesity and hypercholesterolemia.

## Methods

### Animals, diet and experimental set-up

Male C57BL/6 J mice (The Jackson Laboratory, Bar Harbor, Maine, USA) were housed under a 14 h light- 10 h dark cycle at 21–23 °C and had ad libitum access to water during the entire experiment. Mice were fed a ‘Western’ high fat-high sucrose diet (HF-HSD) with 44.6% of kcal derived from fat (of which 61% saturated fatty acids) and 40.6% of kcal derived from carbohydrates (primarily sucrose 340 g/kg diet) (TD.08811, 45%kcal Fat Diet, Harlan Laboratories Inc., Madison, WI, USA) or a normal chow diet (NCD) (V1534-000 ssniff R/M-H, ssniff Spezialdiäten GmbH, Soest, Germany). Losartan (30 mg/kg bodyweight/day) (Cayman Chemical Company, Ann Arbor, MI, USA) was administered via drinking water. Food intake per cage (*n* = 3–4 mice per cage) was measured when renewing the pellets weekly. The drinking water with losartan was renewed daily.

**Fig. 1:**
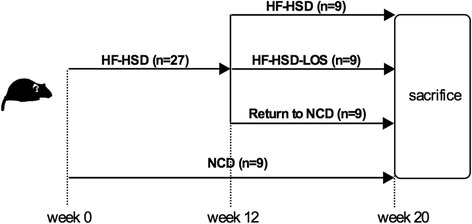
Experimental design. Losartan was given in a dose of 30 mg/kg/day via drinking water

We applied a curative set-up, wherein 27 mice were fed a HF-HSD for 12 weeks (started at age of 6 weeks) to induce the phenotype with NASH, obesity, insulin resistance and hypercholesterolemia, as we published previously [20, 21]. Subsequently, the mice were randomized to the different intervention groups (Fig. 1): 1) HF-HSD group: continuation HF-HSD for the remaining 8 weeks (*n* = 9); 2) HF-HSD-LOS group: continuation HF-HSD together with losartan for the remaining 8 weeks (*n* = 9) and 3) Return to NCD group: switch from HF-HSD to NCD (ad libitum) for the remaining 8 weeks of the experiment (*n* = 9). 9 mice received NCD during the entire experiment (20 weeks) (NCD group). Mean food intake during these last 8 weeks of the experiment was 3.40 g/mouse/day (≈10.37 kcal/mouse/day) for the NCD group, 2.85 g/mouse/day (≈ 13.39 kcal/mouse/day) for the HF-HSD group, 2.74 g/mouse/day (≈ 12.87 kcal/mouse/day) for the HF-HSD-LOS group and 2.88 g/mouse/day (≈ 8.78 kcal/mouse/day) for the Return to NCD group. All procedures were approved by the animal welfare committee of the University of Leuven (protocol number: P088/2011).

### In vivo phenotyping

Body weight was monitored weekly on the same day. Two weeks before sacrifice, intra-peritoneal glucose tolerance test (IPGTT) was performed in 6 h fasted mice. Tail vein glucose levels were measured with a Bayer Contour® glucometer immediately before (time point 0 min) and 15, 30, 60, 90 and 150 min after glucose administration (1 g glucose/kg bodyweight). Insulin resistance was estimated using the Homeostasis Model of Insulin Resistance (HOMA-IR) index: (fasting insulin (ng/ml) X fasting glucose (mg/dl))/405 [22].

### Sacrifice

Sacrifice was performed as described previously [20]. After a 6 h fasting period, mice were anesthetized with sodium pentobarbital (i.p. injection, 50 mg/kg body weight) and killed by blood sampling via cardiac puncture. Plasma was obtained by centrifugation of blood (6000 rpm for 5 min at 4 °C) that was collected in heparinized syringes. Tissues were either snap frozen in liquid nitrogen and together with the plasma stored at -80 °C till further biochemical and molecular analyses or preserved for histological analysis.

### Biochemical analyses

Alanine aminotransferase (ALT) and total cholesterol were measured on 200 μl plasma, obtained through cardiac puncture at sacrifice, via automated procedures. Plasma insulin was measured with a mouse Insulin Elisa Kit (Crystal Chem Inc., Downers Grove, IL, USA). ATP content was measured in liver tissue after homogenization in RIPA lysis buffer (1× PBS, 1% Nonidet P-40, 0.5% sodium deoxycholate, 0.1% SDS, 1 mM sodium orthovanadate (Na_3_VO_4_), 200 mM phenylmethanesulfonylfluoride (PMSF) in isopropanol, protease inhibitor cocktail), using the CellTiter-Glo Luminescent Cell Viability Assay (Promega Corp., Madison, WI, USA) according to the manufacturer’s instructions. 4-Hydroxynonenal (HNE), a byproduct of lipid peroxidation and thus a marker of oxidative stress, was measured following the manufacturer’s protocol (OxiSelect HNE-His Adduct ELISA Kit, Cell Biolabs Inc., San Diego, CA, USA).

### RNA isolation and quantitative RT-PCR

At sacrifice, tissue samples were collected for RNA isolation and stored at -80 °C until analysis. RNA was obtained by a two-step procedure. First, the RNA was isolated using the Trizol method according to the manufacturer’s instructions (Invitrogen Life Technologies, Ghent, Belgium) and was further purified with the RNeasy Kit (Qiagen, Chatsworth, CA, USA). One microgram of cellular RNA was reverse transcribed into cDNA using SuperScript II reverse transcriptase and random hexamer primers (Invitrogen Life Technologies, Ghent, Belgium). The PCR reaction was carried out in a mixture that contained appropriate sense- and anti-sense primers and a TaqMan MGB probe in TaqMan Universal PCR Master Mixture (Applied Biosystems, Foster City, CA, USA) (Col1a1: Mm00801666_g1, TGF-β1: Mm01178820_m1, Timp1: Mm00441818_m1, FAT/CD36: Mm01135198_m1, FATP5: Mm00447768_m1, SREBP-1c: Mm00550338_m1, FAS: Mm00662319_m1, ACC1: Mm01304257_m1, PPAR-α: Mm00440939_m1, CPT1A: Mm01231183_m1, MCAD: Mm01323360_g1, PGC1-α: Mm01208835_m1, SIRT1: Mm00490758_m1, UCP2: Mm00627599_m1, TNF-α: Mm00443260_g1, p62: Mm00448091_m1 and Atg3: Mm00471287_m1. Beta-2-microglobulin was used as housekeeping gene (B2M: Mm00437762_m1). Real-time PCR amplification and data analysis were performed using the A7500 Fast Real-Time PCR System (Applied Biosystems, Foster City, CA, USA). Each sample was assayed in duplicate in a MicroAmp optical 96-well plate. The ΔΔCt-method was used to determine relative gene expression levels.

### Western blot

Liver samples were homogenized with the Precellys 24 tissue homogenizer (Bertin Technologies, distributed by VWR, Leuven, Belgium) in a buffer containing 20 mM Tris-HCl pH 7.6, 10% glycerol, 1% Nonidet P-40, 2 μg/ml aprotinin, 5 μg/ml leupeptin, 0.5 μg/ml pepstatin, 10 mM sodium orthovanadate, 34 μg/ml phenylmethylsulfonyl fluoride, 10 mM sodium pyruvate, 100 mM sodium fluoride and 10 mM EDTA. Coomassie Protein Assay Reagent (Pierce Biotechnology Inc., Rockford, IL, USA) in combination with a standard curve of bovine serum albumin was used to determine the corresponding protein content of the homogenates.

Western blots for autophagic markers were performed as described previously [23]. Primary antibodies were purchased from Abcam, Cambridge, UK (microtubule-associated protein-1 light chain 3 (LC3) and actin) or Novus Biologicals, Littleton, CO, USA (p62). Secondary antibodies were obtained from DakoCytomation, Glostrup, Denmark (horseradish peroxidase-conjugated goat-anti-rabbit or goat-anti-mouse antibodies). Actin expression was used to control for equal loading. Relative protein levels were normalized to the mean of the ratio in the NCD mice.

### Histological analyses

Histological analyses were performed as described previously [20]. Liver samples were routinely fixed in buffered formalin (4%) and embedded in paraffin. Serial 4 μm thick sections were stained with hematoxylin and eosin (H&E) and picrosirius red to assess fibrosis. All liver biopsies were analysed by an expert liver pathologist, blinded to the dietary condition or pharmacological intervention. Steatosis, activity and fibrosis were semi-quantitatively scored according to the NASH-Clinical Research Network criteria [24]. The amount of steatosis (percentage of hepatocytes containing fat droplets) was scored as: 0 (<5%), 1 (5–33%), 2 (>33–66%) and 3 (>66%). Hepatocyte ballooning was classified as: 0 (none), 1 (few) or 2 (many cells/prominent ballooning). Foci of lobular inflammation were scored as 0 (no foci), 1 (<2 foci per 200× field), 2 (2–4 foci per 200× field) and 3 (>4 foci per 200× field). Fibrosis was scored as stage F0 (no fibrosis), stage F1a (mild, zone 3, perisinusoidal fibrosis), stage F1b (moderate, zone 3, perisinusoidal fibrosis), stage F1c (portal/periportal fibrosis), stage F2 (perisinusoidal and portal/periportal fibrosis), stage F3 (bridging fibrosis) and stage F4 (cirrhosis). Diagnosis of NASH was based on accepted histological criteria [25, 26]. Severity of the disease was assessed using the NAS (nonalcoholic fatty liver disease activity score) as the unweighted sum of scores of steatosis, hepatocyte ballooning and lobular inflammation [24]. Percentage of fibrosis was quantitated by morphometry from digitalized sirius red stained sections using the Aperio system after tuning the threshold of fibrosis detection under visual control. Result is expressed as collagen proportional area [27].

### Statistical analyses

Statistical analyses were performed with GraphPad Prism version 6 (GraphPad Software, La Jolla, CA, USA) and JMP10.0.0 (SAS Institute Inc, Cary, NC, USA). Significant differences were estimated by one-way ANOVA and Tukey post-hoc analysis (parametric samples) or Kruskal-Wallis and Dunn’s multiple comparisons test (nonparametric samples). A *P*-value of < 0.05 was considered as statistically significant. Significance is represented by: *: *P* < 0.05, **: *P* < 0.01 and ***: *P* < 0.001. Error bars are standard error of mean (SEM).

## Results

### Dietary intervention normalizes obesity, insulin resistance and hypercholesterolemia

**Fig. 2:**
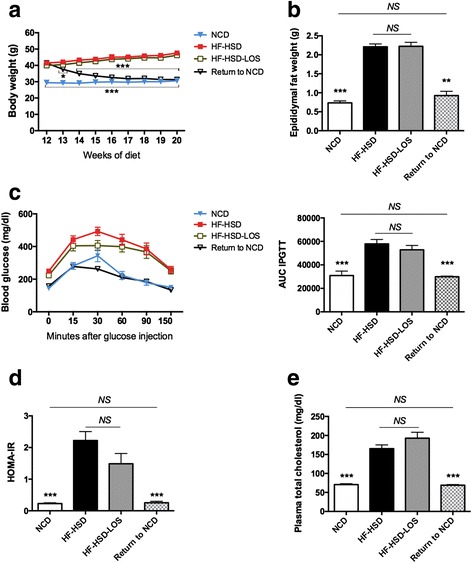
Systemic metabolic effects of dietary intervention (Return to NCD) and losartan (HF-HSD-LOS). **a** Progression of body weight (*n* = 9 per group). **b** Epididymal fat pad weight (*n* = 9 per group). **c** Intra-peritoneal glucose tolerance test (6 h fasting, 1 g/kg glucose) with associated area under the curve values, determined 2 weeks before sacrifice (*n* = 9 for HF-HSD and HF-HSD-LOS mice and *n* = 6 for NCD and Return to NCD mice). **d** HOMA-IR (fasting glucose (mg/dl) X fasting insulin (ng/ml)/405) at sacrifice (*n* = 8 per group). **e** Fasting plasma total cholesterol (*n* = 9 per group). Asterisks indicate significant difference versus HF-HSD mice. Data are presented as mean. Error bars are SEM

Mice in which the HF-HSD was switched to NCD (Return to NCD), displayed a normalization of body weight (Fig. 2a) and epididymal fat pad weight (Fig. 2b). In addition, dietary intervention normalized glucose levels during IPGTT (Fig. 2c), HOMA-IR (Fig. 2d) and plasma total cholesterol levels (Fig. 2e).

On the contrary, losartan did not have an effect on established obesity. Body weight (Fig. 2a) and epididymal fat pad weight (Fig. 2b) were not different between the HF-HSD and HF-HSD-LOS group. Losartan did not affect glucose homeostasis. We observed no significant differences in glucose levels during IPGTT (Fig. 2c) or HOMA-IR (Fig. 2d) between HF-HSD and HF-HSD-LOS mice. Plasma total cholesterol levels were not influenced by losartan (Fig. 2e).

### Dietary intervention, in contrast with losartan, completely reverses pre-existent NASH and fibrosis

**Fig. 3:**
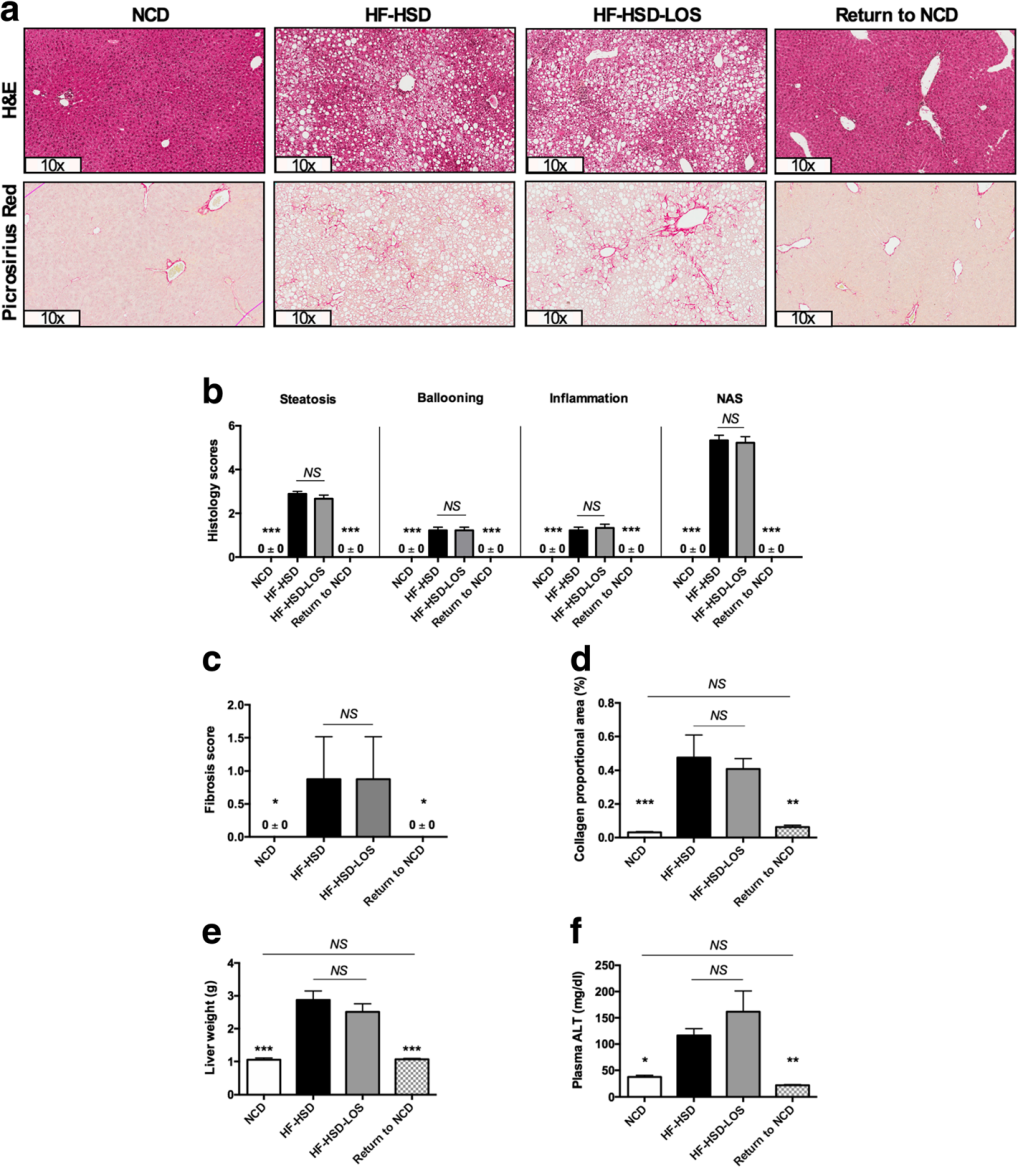
Effects on liver histology of dietary intervention (Return to NCD) and losartan (HF-HSD-LOS). **a** Representative hematoxylin and eosin (H&E) and picrosirius red stained liver histology images per dietary group. **b** Steatosis, hepatocyte ballooning, inflammation scores and NAFLD activity score (NAS). **c** Fibrosis score. **d** Morphometric analysis of collagen proportional area. **e** Liver weight. **f** Plasma ALT. Asterisks indicate significant difference versus HF-HSD mice. *N* = 9 per group for all assays. Data are presented as mean. Error bars are SEM

Remarkably, switch from HF-HSD to NCD in mice with NASH, completely reversed steatosis, hepatocyte ballooning, inflammation and fibrosis (Fig. 3a). Histological scores of Return to NCD mice were identical to that of mice that were fed a NCD during the entire experiment (Fig. 3b and c). In correspondence, morphometric assessment showed a significant decrease of collagen proportional area in Return to NCD mice (Fig. 3d). In addition, liver weight and plasma alanine transaminase (ALT) levels (as a marker of liver damage) were normalized by the dietary intervention (Fig. 3e and f).

Losartan did not improve nor halt the progression of pre-existent NASH (Fig. 3a). Steatosis, hepatocyte ballooning, inflammation and fibrosis scores did not differ between HFD-HSD and HF-HSD-LOS mice (Fig. 3b and c). Collagen proportional area, liver weight and plasma ALT levels were not significantly affected by losartan (Fig. 3d-f).

### Dietary intervention targets several pathways involved in NASH

**Fig. 4:**
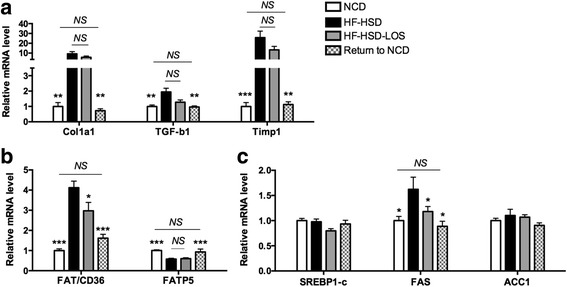
Effect of dietary intervention (Return to NCD) and losartan (HF-HSD-LOS) on fibrogenesis and hepatic lipid metabolism. **a** mRNA levels of genes involved in fibrogenesis (Collagen type I alpha 1 (Col1a1), transforming growth factor β1 (TGF-β1) and tissue inhibitor of metalloproteinase 1 (Timp1)). **b** mRNA levels of genes involved in fatty acid transport (Fatty acid translocase/cluster of differentiation 36 (FAT/CD36), fatty acid transporter member 5 (FATP5)). **c** mRNA levels of genes involved in lipogenesis (Sterol regulatory element binding transcription factor 1 (SREBP1c), fatty acid synthase (FAS), acetyl-CoA carboxylase 1 (ACC1)). Asterisks indicate significant difference versus HF-HSD mice. *N* = 9 per group for all assays. Data are presented as mean. Error bars are SEM

In agreement with the histological findings, dietary intervention (Return to NCD) normalized hepatic mRNA levels of genes involved in fibrogenesis (Col1a1, TGF-β1, Timp1) (Fig. 4a). Furthermore, dietary intervention targets lipid metabolism. mRNA levels of genes involved in fatty acid uptake/transport (FAT/CD36 and FATP5) (Fig. 4b) and lipogenesis (FAS) (Fig. 4c) were normalized in Return to NCD mice.

**Fig. 5:**
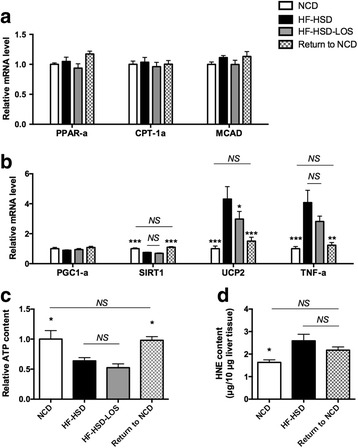
Effect of dietary intervention (Return to NCD) and losartan (HF-HSD-LOS) on mitochondrial parameters. **a** mRNA levels of genes involved in fatty acid β-oxidation: Peroxisome proliferator activated receptor α (PPAR-α), carnitine palmitoyltransferase 1A (CPT1A) and medium chain acyl-Coenzyme A dehydrogenase (MCAD). **b** mRNA levels of genes involved in regulation of mitochondrial function (Peroxisome proliferator-activated receptor gamma coactivator 1-α (PGC1-α), Sirtuin1 (SIRT1), uncoupling protein 2 (UCP2) and tumor necrosis factor-α (TNF-α)). **c** ATP content. **d** HNE concentration as marker of oxidative stress. Asterisks indicate significant difference versus HF-HSD mice. *N* = 9 per group for all assays. Data are presented as mean. Error bars are SEM

At least on mRNA level, we observed no significant differences in fatty acid β-oxidation between the dietary groups at the moment of sacrifice (Fig. 5a). This probably can be explained by the decline of an initial increase of fatty acid β-oxidation in NASH, as we demonstrated previously, which also might be the case after dietary intervention [20]. Dietary intervention affects other parameters involved in mitochondrial function. mRNA levels of SIRT1 (key regulator of mitochondrial function), TNF-α (inducer of mitochondrial dysfunction) and UCP2 (uncouples oxidation from ATP synthesis) were normalized in Return to NCD mice (Fig. 5b). In addition, hepatic ATP levels were restored after dietary intervention (Fig. 5c). Hepatic HNE content (marker of oxidative stress) was not significantly decreased in Return to NCD mice (Fig. 5d). This indicates that oxidative damage by itself is not sufficient to sustain the NASH phenotype in our set-up.

**Fig. 6:**
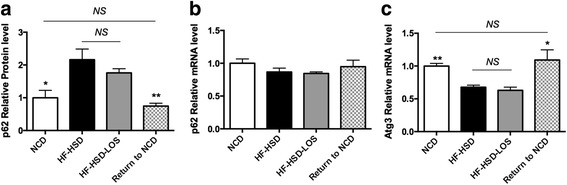
Effect of dietary intervention (Return to NCD) and losartan (HF-HSD-LOS) on hepatic autophagic parameters. **a** Protein levels of p62. **b** mRNA levels of p62. **c** mRNA levels of autophagocytosis associated protein 3 (Atg3). Asterisks indicate significant difference versus HF-HSD mice. *N* = 9 per group for all assays. Data are presented as mean. Error bars are SEM

Hepatic protein levels of p62 (a protein directing ubiquitinated proteins to the autophagic machinery that is known to accumulate when autophagy is impaired or insufficient) were increased in HF-HSD mice (Fig. 6a). In the absence of an increase in p62 mRNA levels (Fig. 6b), the p62 protein accumulation thus suggests an impaired autophagic flux [23, 28]. Disturbed autophagy in HF-HSD mice is further supported by reduced mRNA levels of Atg3 (Fig. 6c). Dietary intervention abolished the p62 protein accumulation and returned Atg3 mRNA to normal levels, suggesting restoration of the autophagic flux (Fig. 6a-c). The LC3-II/LC3-I ratio, which is used as a marker of mature autophagosome formation, was not affected by HF-HSD or the dietary intervention (data not shown).

Finally, losartan significantly decreased mRNA levels of FAT/CD36, FAS and UCP2, but did not affect any other of the aforementioned parameters (Figs. 4, 5 and 6).

## Discussion

In this study, dietary intervention completely reversed all features of pre-existent NASH, together with obesity, insulin resistance and hypercholesterolemia in a representative mouse model. Losartan had no effect on these established metabolic changes.

The beneficial effect of life style intervention on NASH, focused on both dietary and exercise habits, has been demonstrated in only a few randomized controlled trials with histological endpoints [8, 10, 11]. In addition, human studies are limited in time and do not allow clear differentiation of the dietary effects relative to physical activity [7, 10]. Moreover, only 30 to 40% of patients achieve target weight loss even in trials implementing an intensive life style program including behavorial strategies, which reflects the variable patient adherence [8, 10, 11, 29]. Therefore, we opted to use a relevant NASH mouse model to explore the full therapeutic potential of dietary intervention, which additionally allowed us to study the hepatic mechanisms behind it. To our knowledge, our dietary intervention represents the most effective therapy for NASH (i.e., complete resolution) demonstrated in rodents. Dietary intervention also resolved fibrosis in our model. This is an important finding since fibrosis is the major determinant for progression to end-stage liver disease and thus represents a main target to combat in NASH [20, 30, 31]. In 3 recent studies, a comparable dietary intervention (switch from a high fat diet to a NCD) was applied in obese mice with NASH [12, 32, 33]. However, the beneficial effects on liver histology were less pronounced, likely due to the shorter length of the dietary intervention (3 weeks) in one study and the use of genetically modified mice (hyperphagic *foz/foz* (*Alms1* mutant) and low-density lipoprotein receptor knock out mouse model) in the two other studies [12, 32, 33].

The fact that dietary intervention affects the precipitating insult and in addition has the ability to normalize several molecular pathways involved in NASH, probably explains its superior efficacy compared with pharmacological agents that mostly only target a single or limited number of relevant pathways. We showed that dietary intervention targets pathways involved in fibrogenesis, lipid metabolism, mitochondrial function and autophagy, together contributing to the beneficial effect on histological level. The time point of sacrifice was 8 weeks after return to NCD. The study of earlier time points may clarify the possibility that a burst of clean-up of accumulating lipids by fatty acid oxidation and possibly autophagy took place in earlier stages after the dietary switch.

In contrast with our animal study, life style interventions (including dietary counselling) yield rather disappointing results in daily patient care. The lack of a well-organized multidisciplinary therapeutic approach from physician’s side probably reinforces this phenomenon [34, 35]. Therefore, in order to exploit the maximal therapeutic potential of dietary intervention, improving patient adherence, possibly by cognitive behaviour therapy embedded in structured programs, should be a main goal in the care of NASH patients in the coming years [35]. The lack of effective pharmacological alternatives and the additional beneficial effect of life style measures on other components of the metabolic syndrome further support this strategy.

However, more insight is needed in the role of specific dietary components in NASH treatment strategies. The evidence for the harmful effects of saturated fatty acid and sucrose intake is solid, whereas the preventive or even therapeutic capacity of specific dietary components (i.e., nutraceutical products) is less clear in the context of NASH [36, 37]. Animal studies using ingredient matched control diets or well-designed human interventional studies could provide further insight. This might potentiate the use of these molecules as therapeutics and explain the possible beneficial effects of the Mediterranean diet in NAFLD/NASH [36, 37]. Another issue that remains to be addressed is the influence of genetic variation in humans (in contrast with the inbred C57BL/6 J mouse strain we used for our experiments) on treatment outcome. Very little is known about the effect of genetics on the response to dietary or pharmacological treatment of NASH [38]. It was recently shown that genetic variation in PNPLA3 (adiponutrin) indeed confers sensitivity to weight loss-induced decrease of liver fat in humans [39]. Therefore, identifying (genetic) predictors for response to treatment could potentiate an individualized approach, stimulate patients to persevere the necessary dietary changes and increase their physical activity, and may thus further improve clinical outcome [38].

In parallel, we explored the therapeutic potential of losartan. Losartan seems an attractive candidate for the treatment of NASH, because it is a safe drug widely used for the treatment of hypertension (which is a part of the metabolic syndrome) and it targets angiotensin II that is thought to play an important role in NASH [14, 40]. However, the few available preliminary human and animal studies yielded equivocal results [14–19]. In addition, losartan has only been tested in normal or hyperphagic Otsuka Long-Evans Tokushima fatty (OLETF) rats, which were fed a non-physiological choline-deficient L-amino acid-defined diet [16, 18]. In our representative HF-HSD model, losartan did not have a beneficial effect on established NASH, obesity and insulin resistance. At the molecular level, losartan only significantly decreased FAT/CD36 and FAS mRNA together with UCP2 mRNA, but these effects appear insufficient to impact hepatic steatosis or ATP content respectively. We used a losartan dose comparable to the dose used in previous rodent studies on the metabolic and cardiovascular effects of losartan [41]. It may be that the duration of losartan administration in our experimental set-up was not long enough, although the same intervention period was largely sufficient to resolve NASH and associated systemic metabolic features by dietary intervention. However, in contrast with the dietary switch group, losartan had to counteract the effects of the continued HF-HSD, mimicking the human obesiogenic environment. It might be interesting to explore whether higher losartan doses, a longer administration time or losartan in combination with dietary intervention or another drug would be effective in NASH resolution. Possibly, a better effect could be achieved by using telmisartan, because of its combination of peroxisome proliferator-activated receptor (PPAR)-γ and angiotensin receptor blocking activity [42, 43]. Taken together, our pre-clinical data do not support the use of losartan to treat NASH.

## Conclusions

In contrast with losartan, dietary intervention completely restores the metabolic phenotype, on both hepatic and systemic level, in mice with pre-existent NASH, obesity, insulin resistance and hypercholesterolemia. Our mouse model and set-up represent a useful tool to further study the effects of dietary and pharmacological interventions in NASH and the metabolic syndrome. In addition, our results emphasize the need to identify the pathophysiological and therapeutic barriers that preclude similar results in the current treatment of human NASH. This would facilitate the development of effective strategies to help patients to implement and maintain a healthy diet style in order to treat NASH and other components of the metabolic syndrome.

## Abbreviations

ACC1: Acetyl-CoA carboxylase 1
ALT: Alanine transaminase
Atg3: Autophagocytosis associated protein 3
ATP: Adenosine triphosphate
B2M: Beta-2-microglobulin
Col1a1: Collagen type I alpha 1
CPT1A: Carnitine palmitoyltransferase 1A
FAS: Fatty acid synthase
FAT/CD36: Fatty acid translocase/cluster of differentiation 36
FATP5: Fatty acid transporter member 5
H&E: Hematoxylin and eosin
HF-HSD: High fat - high sucrose diet
HNE: 4-hydroxynonenal
HOMA-IR: Homeostasis model of insulin resistance
i.p.: Intra-peritoneal
IPGTT: Intra-peritoneal glucose tolerance test
LC3: Microtubule-associated protein-1 light chain 3
MCAD: Medium chain acyl-Coenzyme A dehydrogenase
NAFLD: Non-alcoholic fatty liver disease
NAS: NAFLD activity score
NASH: Non-alcoholic steatohepatitis
NCD: Normal chow diet
OLETF: Otsuka Long-Evans Tokushima fatty
PGC1-α: Peroxisome proliferator-activated receptor gamma coactivator 1-alpha
PNPLA3: Patatin-like phospholipase domain-containing protein 3
PPAR-α: Peroxisome proliferator activated receptor alpha
PPAR-γ: Peroxisome proliferator-activated receptor gamma
RAS: Renin-angiotensin system
SIRT1: Sirtuin1
SREBP1c: Sterol regulatory element binding transcription factor 1
TGF-β1: Transforming growth factor beta 1
Timp1: Tissue inhibitor of metalloproteinase 1
TNF-α: Tumor necrosis factor-alpha
UCP2: Uncoupling protein 2
